# Effect of ultra violet irradiation on the interplay between Th1 and Th2 lymphocytes

**DOI:** 10.3389/fphar.2015.00056

**Published:** 2015-03-24

**Authors:** Salma Y. Abo Elnazar, Amany A. Ghazy, Hossam E. Ghoneim, Abdul-Rahman M. Taha, Amira M. Abouelella

**Affiliations:** ^1^Department of Immunology, Medical Research Institute, Alexandria UniversityAlexandria, Egypt; ^2^Department of Medical Biophysics, Medical Research Institute, Alexandria UniversityAlexandria, Egypt; ^3^Radiation Biology Department, National Centre for Radiation Research and Technology, Atomic Energy AuthorityCairo, Egypt

**Keywords:** UV radiation, lymphoproliferative response, polyclonal mitogens, Th1/Th2 response

## Abstract

Although ultraviolet (UV) radiation is used to treat several types of diseases, including rickets, psoriasis, eczema, and jaundice, the prolonged exposure to its radiation may result in acute and chronic health effects particularly on the skin, eyes, and the immune system.

**Aim**: This study was carried out to show the effect of UV on both of the lymphoproliferative response and their capacity to produce IL-12 and IL-10 in mice.

**Methods**: Mice were exposed to whole body UVB and tested for the effect of recovery times on lymphocyte proliferation and cytokine production. In addition, direct irradiation of spleens and lymphocyte suspension was carried out. Basal and mitogens-stimulated lymphocyte proliferation was assessed by MTT assay while IL-10 and IL-12 were measured using ELISA.

**Results**: There was a significant suppression in lymphocyte proliferation in comparison with control. IL-12 level was significantly reduced while the level of IL-10 was increased. Con A and PWM mitogens had no significant changes in IL-10 while Con A caused a highly significant increase in IL-12 at day 6 of recovery in UVB body irradiation.

**Conclusion**: Exposure to UVB radiation could cause a state of immune suppression and shifts Th1/Th2 cell response. This effect is closely associated with the reduction of Th1 cytokines’ expression and increase in Th2 cytokines’ levels.

## Introduction

Ultraviolet (UV) spectrum includes short-wave (UVC; 200–290 nm), mid-wave (UVB; 290–320 nm), and long-wave (UVA; 320–400 nm) components of the radiation. UVB radiation is proved to be a mutagen, and has been shown to be responsible for sunburn, oxidative stress, and immune suppression. Notably, decreases in the stratospheric ozone layer are permitting more UVB radiation to reach the Earth’s surface (Katiyar, 2007).

Photoimmunology is a term defined as the study of the effects of non-ionizing radiation on the immune system. It grew from experiments designed to understand the mechanism(s) underlying UVB-induced skin carcinogenesis (Ullrich and Byrne, 2012). Numerous studies in the field of Photoimmunology have tried to identify the biological impact of UV on the mammalian immune system. It is reported that the immune suppressive effects of solar radiation are mediated mostly by UVB (Schwarz, 2005).

Ultraviolet radiation can suppress the immune system in several ways. The UVB spectrum inhibits antigen presentation, induces release of immunosuppressive cytokines, causes apoptosis of leukocytes and elicits DNA damage which is a molecular trigger of UV-mediated immunosuppression (Ullrich, 2005; Katiyar, 2007). However, UVB does not cause general immunosuppression but rather inhibits immune reactions in an antigen-specific fashion. This specific immunosuppression is mediated by antigen-specific suppressor/regulatory T cells (Schwarz, 2005; Katiyar, 2007).

Activated naïve CD4^+^ T-cells differentiate into T-helper 1 (Th1) under the influence of interleukin 12 (IL-12) produced by dendritic cells (DCs) and macrophages. Numerous studies illustrated the importance of IL-12 for the development of protective innate and adaptive responses (Jacobo et al., 2011). In contrast with Th1 cells, the differentiation of naïve CD4^+^ T-cells into Th2 cells is potentially promoted by IL-4 produced by DCs and mast cells. The type of antigens initiating DCs response appears to be a key factor in determining whether Th1 or Th2 lineage will develop (Guo et al., 2009).

It has been demonstrated that ionizing and non ionizing radiations have the potential of interrupting the balance of Th1/Th2 cell response; an effect that is closely associated with the reduction of Th1 cytokine expression and increase in Th2 cytokines’ levels. This radiation induced alteration in Th1/Th2 balance is profoundly related to the modulation of cytokine-mediated transcriptional factors and signaling pathways. Accordingly, enhanced Th2 response may mediate immune suppression and reduce, to some degree, the protective arm of cellular immunity (Yoshiki et al., 2012).

It is reported that UV radiation stimulates cells to release immune suppressive soluble mediators, including IL-10, which is secreted mainly by Th2 (Ullrich, 2005). These soluble mediators can enter the circulation and suppress the immune system in a systemic manner. The immunosuppressive effects of IL-10 may be one of the mechanisms by which tumors may escape immunologic control (Vaid et al., 2013).

The immune-regulatory IL-12 has been shown to exhibit the capacity to reduce the UV-induced DNA damage, immunosuppression and apoptosis (Schwarz et al., 2002, 2005). IL-12 has been shown to prevent the development of regulatory T cells and even break UV-induced tolerance by un-identified mechanisms (Schwarz et al., 2005).

The present study aimed to investigate the effects of non-ionizing UVB energy on the splenic lymphocytes proliferation and their capacity to release IL-12 and IL-10 as indicators for Th1 and Th2 responses, respectively.

## Materials and Methods

### Experimental Animals

This study was conducted on 80 male inbreed C57BI/6j mice aging 6–8 weeks and weighing 20–25 g each. The animals were purchased from Theodor Bilharz Research Institute, Cairo, Egypt. This study was approved by the Ethics Committee of Medical Research Institute, Alexandria University. All experiments conform to the regulatory standards of Medical Research Institute, Alexandria University. All reagents used in the study were supplied by Sigma–Aldrich Chemical (St Louis, MO, USA) unless otherwise stated.

### UVB Irradiation

Mice were divided into two main groups; the first group was composed of 60 mice. They were subjected to UV light in the form of UVB (100 mJ/cm^2^) for 1 h at a fixed distance of 25 cm, following the original protocol described by Hammerberg et al. (1996) and Kimura et al. (1999) with slight modifications. Prior to UVB irradiation, the dorsal skin of mice was clipper-shaved. UV lamp was purchased from (TLD 10W X2, AMIR AM-4020W, China), the great majority of the resulting wavelengths were in the UVB range (290–320 nm, ∼95%) with peak emission at 314 nm. This group was subdivided into three groups (20 mice/group) according to lymphocytes stimulation; either un-stimulated or Con A or PWM-induced cells. The second group was composed of 20 age and sex-matched non UVB irradiated mice as a control group.

In addition to the whole body irradiation, direct irradiation of isolated lymphocyte suspensions (at a concentration of 10 × 10^6^) was performed to test the direct effect of irradiation on our study parameters.

### Animal Scarification

After whole body irradiation, animals were sacrificed at three time intervals (represented zero, 3 and 6 days) post irradiation. Six to seven mice were sacrificed at each time interval from each group.

### Preparation of Splenocyte Suspension

Spleens were collected separately into sterile plastic petri-dishes (7 × 1.5 cm) containing 50 mM phosphate buffer saline (PBS), pH 7.2. Lymphocytes of spleen were prepared individually from excised spleens, according to the method originally employed by Coligan et al. (1994) under strictly aseptic conditions. Single cell suspension was obtained from each preparation by simple sedimentation to get rid of splenic tissue debris and large cell aggregates. Contaminating RBCs were lysed out by suspending cell pellets in 5 ml sterile ammonium chloride solution (0.83%) for 5 min Lymphocytes of spleen were washed twice with 50 mM PBS, pH 7.2 and finally suspended in the tissue culture media RPMI-1640 (Sigma–Aldrich, St. Louis, MO, USA) supplemented with L-glutamine, 10% heat inactivated fetal calf serum, penicillin (100 IU/ml) and streptomycin (100 μg/ml). Single cell suspensions were subsequently employed in tissue cultures.

### Cell Viability Testing

Prepared lymphocytes of spleen were tested for viability by trypan blue dye exclusion technique (Castro-Concha et al., 2005). Volumes of 10 μl of each cell suspension was gently mixed with equal volumes of 0.2% trypan blue dye and left for 2–5 min at room temperature. Aliquots were examined microscopically in a hemocytometer. Viable cells are characterized by unstained cytoplasm and shiny boundaries whereas non-viable cells have blue cytoplasm and undefined boundaries. The percentage of the viable cells was estimated for each preparation according to the following formula: % viability = (number of viable cells/total cell number) × 100. Then cell counts were adjusted at 2 × 10^6^ cells/ml for subsequent tissue culture.

### Assessment of Cell Proliferation by MTT Assay

The proliferative functions of isolated lymphocytes of spleen were monitored by the *in vitro* mitogenic polyclonal activation using two different plant lectins as mitogens; concanavalin A (Con A) and Pockweed (PWM) adopting the standard protocol of Bieback et al. (2003) with minor modifications. For assessment of the state of lymphocytes proliferation following Con A and PWM, the tetrazolium compound MTT [3-(4,5-dimethylthiazol-2-yl)-2,5-diphenyltetrazolium bromide] was added to cultured cells. MTT is reduced by metabolically active cells to insoluble purple formazan dye crystals. Detergent is then added, destructing cell membranes and solubilizing the crystals so that the absorbance can be read using a spectrophotometer. The rate of tetrazolium reduction is directly proportional to the rate of cell proliferation. Thus responsive lymphocytes will have higher absorbance while weakly activated and non-responsive cells will demonstrate lower optical densities reflecting only their basal metabolic state (Riss and Moravec, 1992).

Volumes of 100 μl of supplemented RPMI-1640 tissue culture medium were dispensed into rows of nine wells within 96 wells flat-bottomed microtiter tissue culture plates (Greiner bio-one, Germany). Volumes of 100 μl of each lymphocyte suspension were dispensed in triplicates at final concentration of 2 × 10^6^ cells/ml. Then two groups of wells were pulsed separately with 2 μl of Con A or PWM at a final concentration of 10 μl/ml. The remaining group was left without mitogen reflecting the basal metabolic activity of the respective lymphocyte suspension. The plate was incubated at 37°C in humidified CO_2_ incubator (5% CO_2_ and 95% O_2_) for 2 days. At the end of the culture, 10 μl of MTT reagent (Cayman chemical company, Germany) was added to all wells and mixed gently then the plate was incubated for 3–4 h. At the end of incubation period formazan deposits produced within the cells, subsequently 100 μl of the ready to use crystal-dissolving solution was added to each well producing homogenous dark blue to purple solution with different intensities. Finally, the optical density (OD) of each well was measured at 570 nm using a microplate reader (Bio-Rad, Hercules, CA, USA). The average values from triplicate readings of Con A and PWM stimulated versus un-stimulated wells were determined and used to calculate the stimulation index (SI; Singh et al., 2012) as follow: SI = mean of OD values of mitogen stimulated wells/mean of OD values of un-stimulated wells.

### Induction of Cytokine Production and Assessment of T Helper (Th) Bias

The *in vitro* capability of splenic lymphocytes to release IL-10 and IL-12 cytokines, representative for Th2 and Th1 cells, respectively, was evaluated by using short term culture. This was monitored either spontaneously or in the presence of Con A or PWM mitogens and was taken as a monitor for assessment of Th bias following exposure to non-ionizing radiation. Details of the tissue culture protocol were adopted from the standard method developed by Davis (1995).

Volumes of 1 ml of lymphocyte suspension (2 × 10^6^ cells/ml of supplemented RPMI-1640) corresponding to each of the control and UVB-irradiated mice were dispensed in triplicates into 24 wells flat-bottomed microtiter tissue culture plates (Nunclon, Denmark). One group of wells was left without mitogen while the other two were pulsed separately with 10 μl of either Con A or PWM at a final concentration of 10 μl/ml. The plate was then incubated at 37°C in humidified CO_2_ incubator (5% CO_2_ and 95% O_2_) for 2 days. At the end of the culture period, the content of each well was collected and centrifuged at 1800 rpm for 10 min at room temperature. Supernatants were collected and stored at -80°C until measurements of released cytokines.

### Measurement of IL-10 and IL-12

Murine IL-10 and IL-12 were assayed in culture supernatants corresponding to all mice under the study. This was carried out by enzyme linked immunosorbant assay (ELISA) using a commercially available kit from Ray Biotech following the instructions of the manufacturer.

### Statistical Analysis

The data were analyzed using statistical package for social science (SPSS) program version 17.7. Student’s *t*-test was used to compare between the different groups and Paired *t*-test was used for comparison applied to the same group. We adjusted the alpha level of the statistical comparisons by dividing 0.05 by number of comparisons.

## Results

### Effect of UVB Whole Body Irradiation

After UVB irradiation of whole body, mice were left to recover for zero, three and 6 days before being sacrificed and dissected in parallel to control non-irradiated mice. Lymphocytes of spleen were prepared and maintained in short term tissue cultures in either presence or absence of Con A and PWM as T-and B-lymphocyte mitogens, respectively.

#### Effect of UVB Irradiation on Cell Proliferation

**FIGURE 1:**
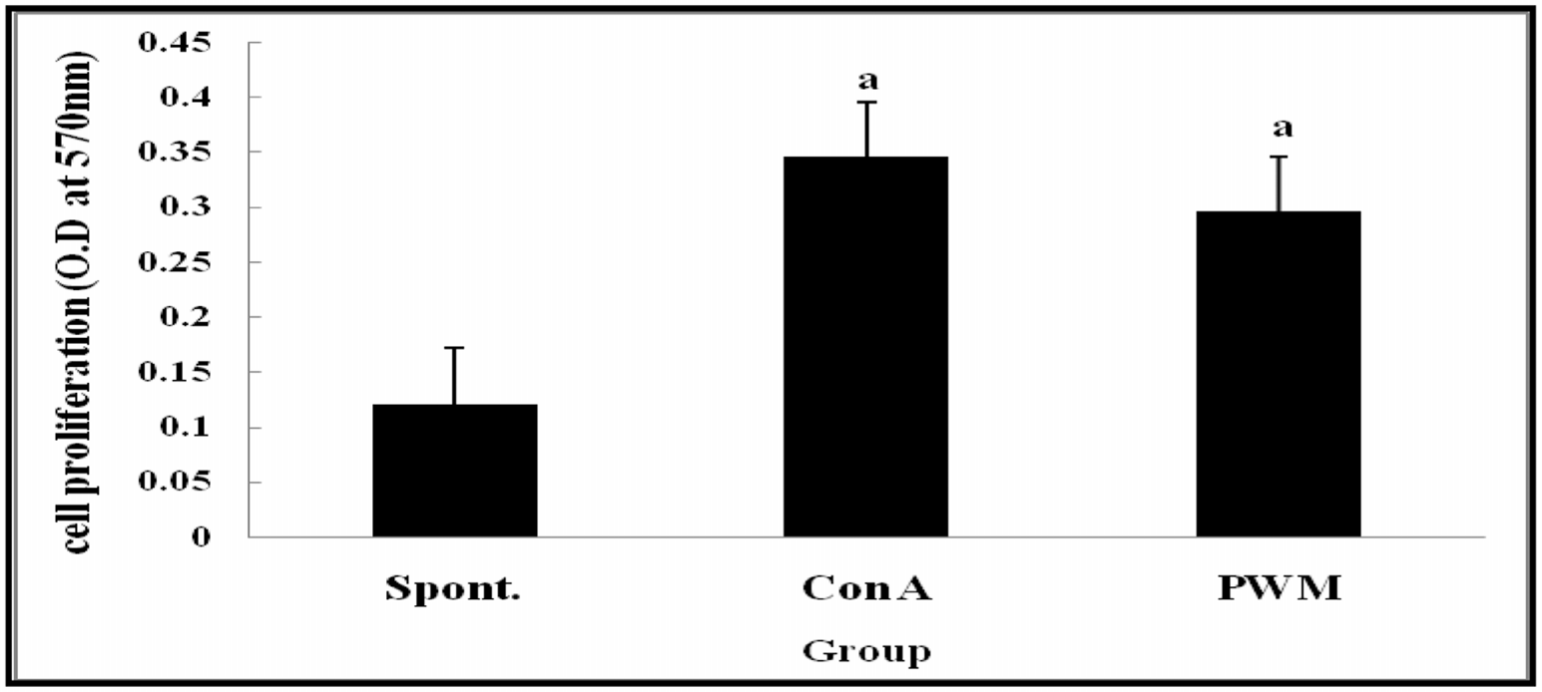
**Cell proliferation in non-irradiated mice showing effect of Con A and PWM.** ^a^Is level of significance between spontaneous (spont) and mitogen induced data.

Statistical analysis of results of non-irradiated (control) mice showed a highly significant elevation in lymphocyte proliferation following *in vitro* stimulation by Con A and PWM than their corresponding un-stimulated cells (**Figure 1**). There was no significant difference between Con A- and PWM-induced proliferation (*p* = 0.441).

**FIGURE 2:**
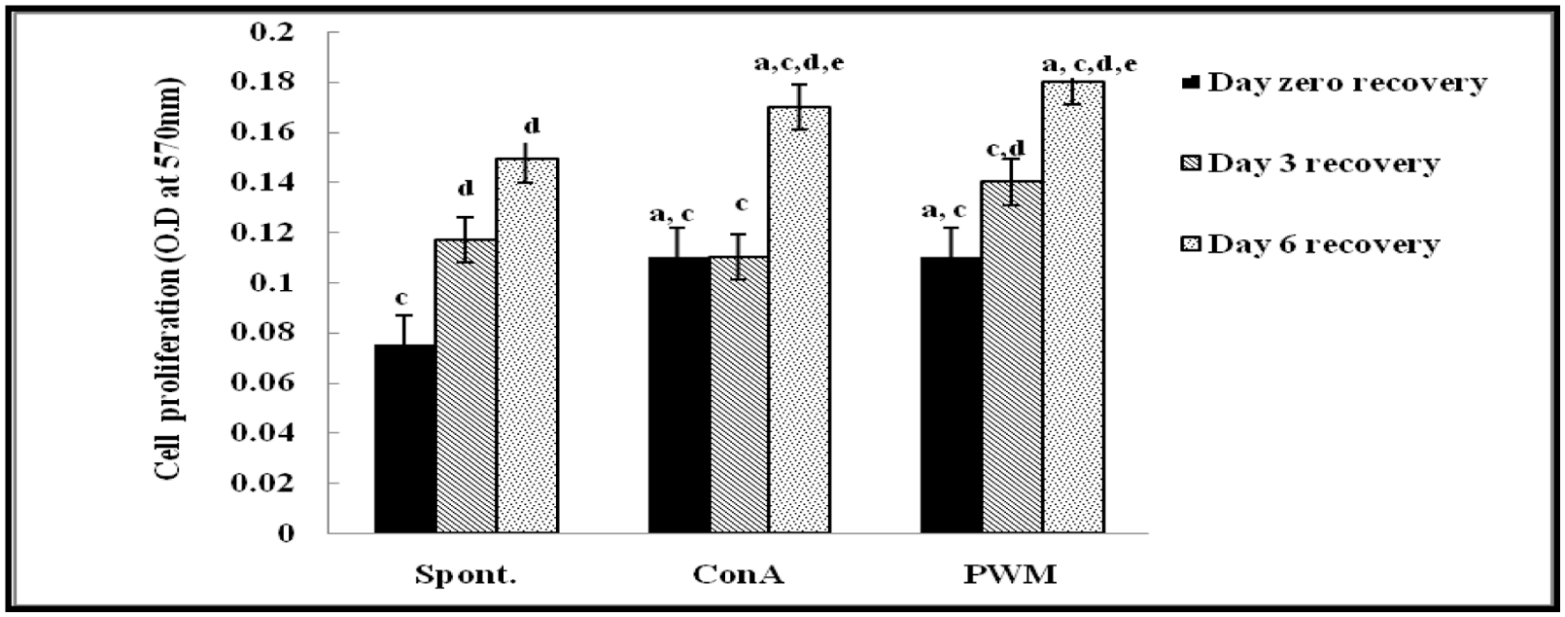
**Cell proliferation in UVB-irradiated mice.** ^a^Level of significance between spontaneous and *in vitro* mitogen induced data, ^b^level of significance between Con A and PWM-induced data, ^c^level of significance between control and UVB irradiation data, ^d^level of significance between days 0, 3, and 6 of recovery, ^e^level of significance between days 3 and 6 of recovery.

Regarding UVB-irradiated mice, statistical analysis of results at 0 day of recovery showed a significant elevation in Con A and PWM-induced lymphocyte proliferation relative to un-stimulated irradiated cells. On the contrary, results showed significant suppression in cell proliferation relative to the non-irradiated control data (**Figure 2**).

At 3 days of recovery, there was no significant change in cell proliferation between mitogen-induced and un-stimulated irradiated cells (**Figure 2**). Relative to results of non-irradiated control mice, there was a significant suppression of cell proliferation except for un-stimulated cells. When comparing between data collected at 0 and 3 days of recovery, basal and PWM-induced proliferation was found to be ameliorated while Con A-induced proliferation was unchanged (**Figure 2**).

At 6 days of recovery, there was significant change between basal and mitogen-induced cell proliferation (**Figure 2**). Relative to results of control mice, there was a significant decrease in mitogen-induced proliferation although spontaneous proliferation was unchanged. When statistical comparison was carried out between data collected at 0, 3, and 6 days of recovery, a significant elevation in basal and mitogen-induced cell proliferation was observed (**Figure 2**).

#### Effect of UVB Irradiation on Cytokine Production

**FIGURE 3:**
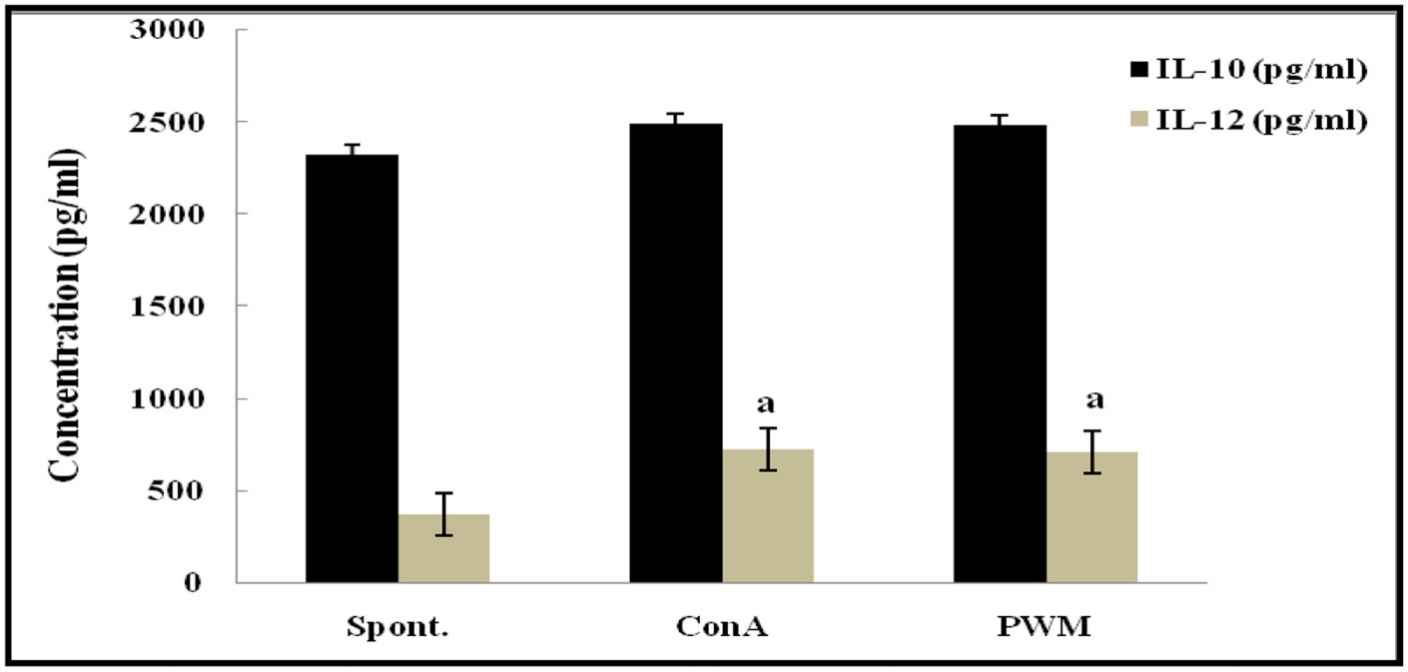
**IL-10 and IL-12 concentrations in non-irradiated mice showing the effect of Con A and PWM.** ^a^Is level of significance between spontaneous and mitogen induced data.

Statistical analysis of non-irradiated (control) mice results showed a highly significant elevation in the capacity to release IL-12 following *in vitro* stimulation by Con A and PWM than their corresponding un-stimulated cells. However, IL-10 release capacity remained unaffected by stimulation (**Figure 3**). There was no significant difference between Con A- and PWM-induced IL-10 or IL-12 release (*p* = 0.963 and 0.870, respectively).

**FIGURE 4:**
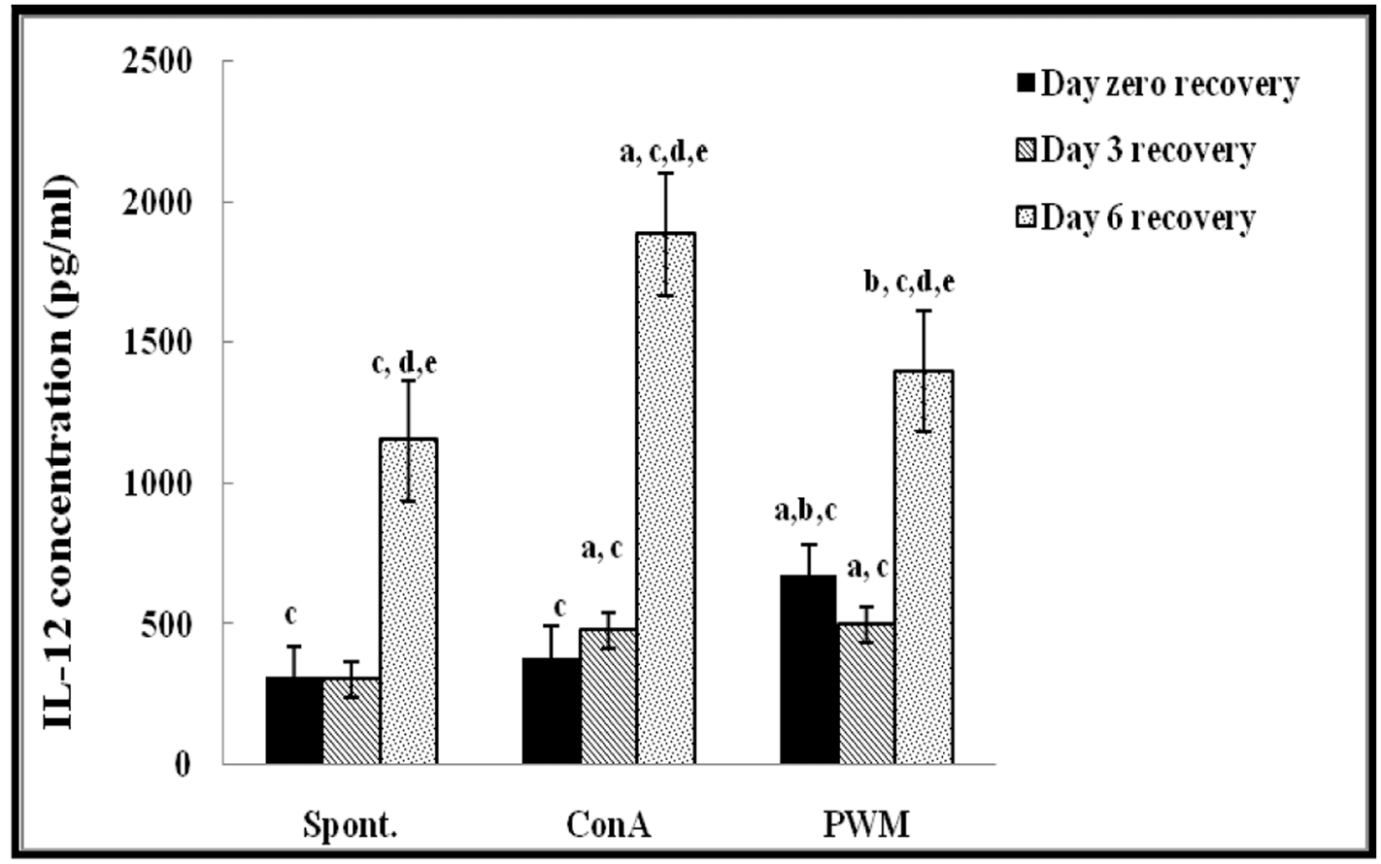
**IL-12 concentraion in UVB-irradiated mice.** ^a^Level of significance between spont. and *in vitro* mitogen induced data, ^b^level of significance between Con A and PWM-induced data, ^c^level of significance between control and UVB irradiation data, ^d^level of significance between data of days 0, 3, and 6 of recovery, ^e^level of significance between data of days 3 and 6 of recovery.

**FIGURE 5:**
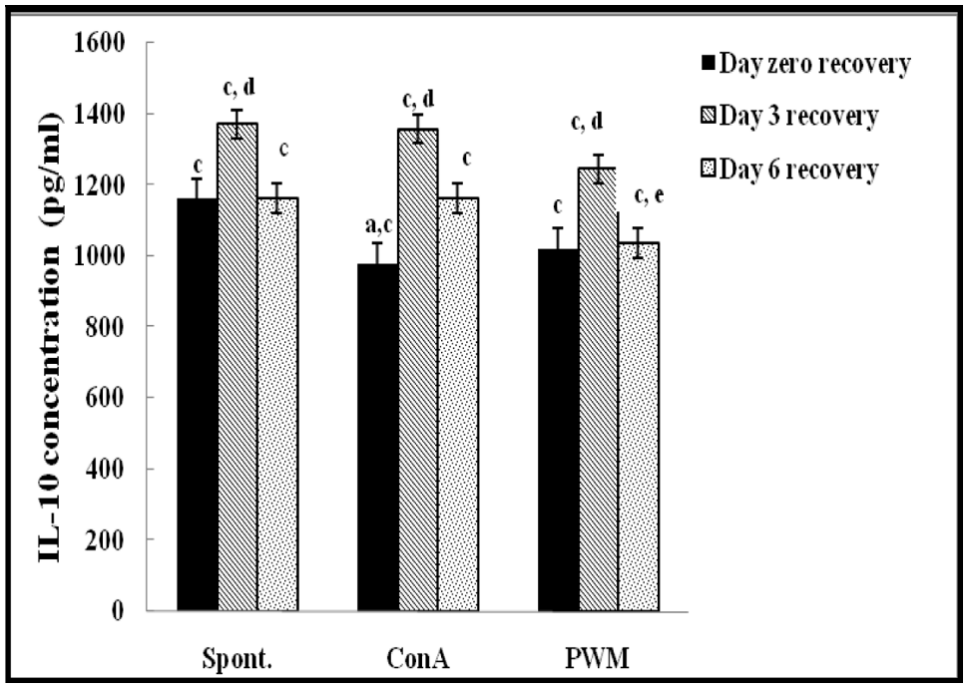
**IL-10 concentraion in UVB-irradiated mice.** ^a^Level of significance between spontaneous and *in vitro* mitogen induced data, ^b^level of significance between Con A and PWM-induced data, ^c^level of significance between control and UVB irradiation data, ^d^level of significance between data of days 0, 3, and 6 of recovery, ^e^level of significance between data of days 3 and 6 of recovery.

Regarding UVB-irradiated mice, statistical analysis of results at 0 day of recovery showed a significant increase in PWM-induced IL-12 release capacity relative to both un-stimulated and Con A-induced cells (*p* < 0.001; **Figure 4**). There was a significant suppression in Con A-induced IL-10 release compared to un-stimulated cells (**Figure 5**). Relative to control data, IL-12 release was significantly suppressed and IL-10 release was significantly increased when mice exposed to UV irradiation without recovery time (**Figure 4**).

At day 3 of recovery, IL-12 release capacity induced by both mitogens was significantly augmented when compared to un-stimulated cells (**Figure 4**). There was no significant change in IL-10 release between mitogens-induced and un-stimulated cells (**Figure 5**). Relative to control data, results of IL-12 release were significantly suppressed, except for un-stimulated cells while IL-10 release was significantly increased in all groups.

At day 6 of recovery, mitogens-induced IL-12 release was significantly increased (**Figure 4**). There was no significant change between basal and mitogen-induced IL-10 release (**Figure 5**). Relative to results of control mice, there was a significant massive production of spontaneous and mitogens induced IL-12 and suppression of IL-10 release.

### Effect of UVB Irradiation of Isolated Splenic Lymphocytes

Following UVB irradiation of lymphocyte suspension, cells were left to recover for either 30 min or 1 h.

#### Effect of UVB Exposure on Cell Proliferation

**FIGURE 6:**
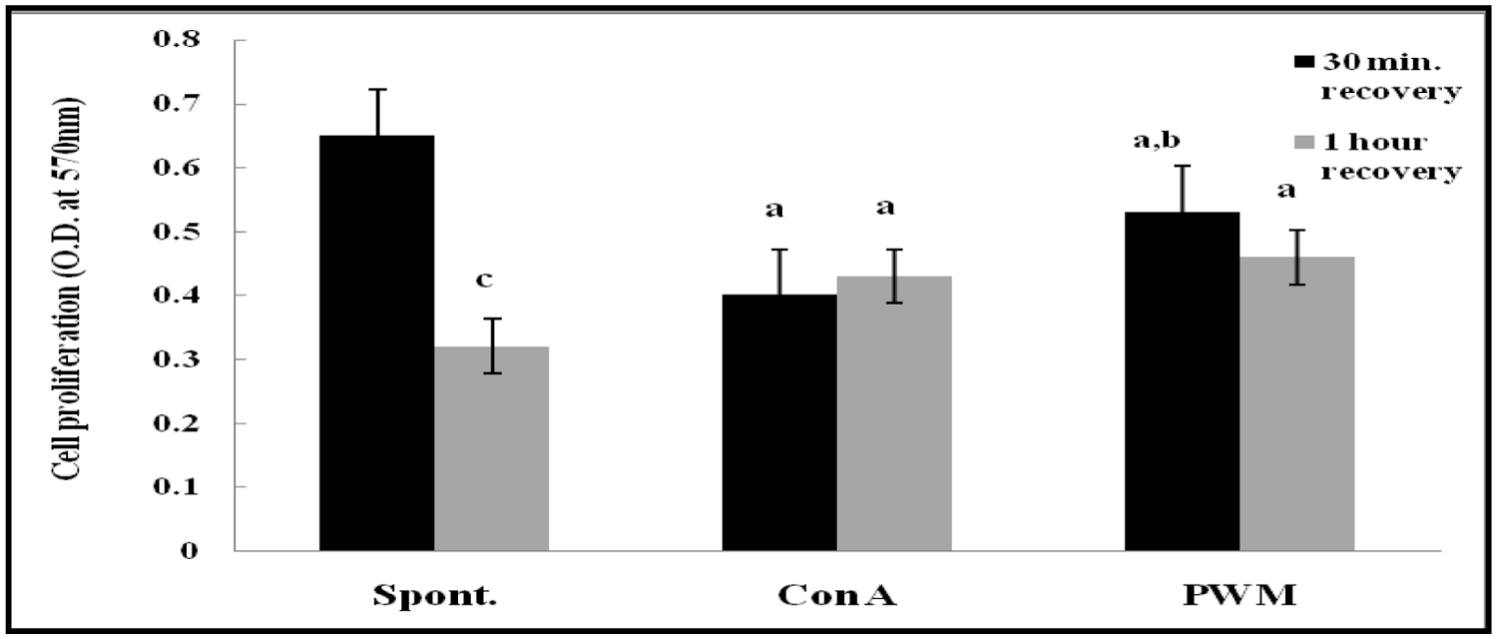
**Cell proliferation either spontaneously or after *in vitro* stimulation with Con A or PWM in UVB irradiated splenic lymphocytes suspension.** ^a^Level of significance between spontaneous and *in vitro* mitogen induced data, ^b^level of significance between Con A-and PWM-induced data, ^c^level of significance between 30 min and 1 h of recovery data.

After 30 min of recovery of lymphocyte suspensions, there was a highly significant decrease in proliferation of mitogen-induced lymphocyte compared to un-stimulated cells (*p* < 0.001 and *p* = 0.029, respectively). Although PWM showed significant increase compared to Con A (*p* = 0.017; **Figure 6**). After 1 h of recovery, similar statistical records were obtained (*p* < 0.001 and *p* = 0.0313, respectively). In addition, there was a highly significant reduction in basal lymphocyte proliferation as compared to that obtained after 30 min of recovery (*p* < 0.001). However no significant difference was obtained between Con A-and PWM-induced proliferation (*p* = 0.291 and *p* = 0.127, respectively; **Figure 6**).

#### Effect of UVB Exposure on Cytokine Production

**FIGURE 7:**
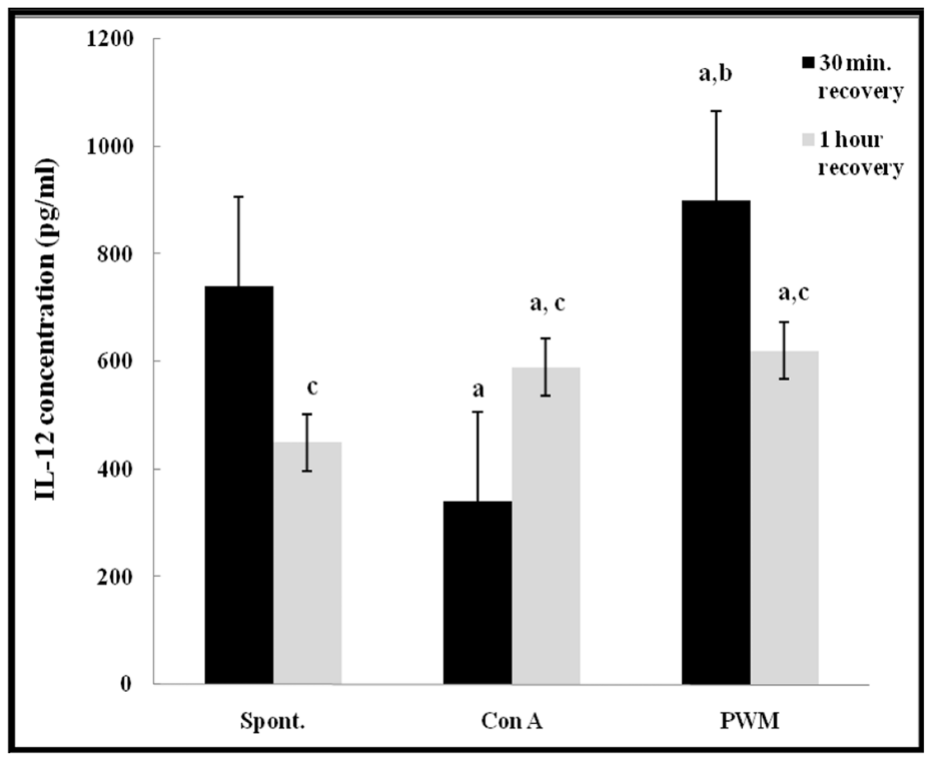
**IL-12 release either spontaneously or after *in vitro* stimulation with Con A or PWM in UVB irradiated lymphocytes suspension. ^a^Level of significance between spontaneous and *in vitro* mitogen induced data.** ^b^level of significance between Con A-and PWM-induced data. ^c^level of significance between 30 min and 1 h recovery data.

After 30 min of recovery, there was a highly significant impairment in IL-12 release from Con A-stimulated cells (*p* < 0.001). PWM induced IL-12 release showed a significant increase as compared to both basal and Con A-stimulated production (*p* = 0.002 and *p* < 0.001, respectively). After 1 h of recovery, there was a significant increase in both Con A- and PWM-induced IL-12 release capacity (*p* = 0.001 and *p* < 0.001, respectively). Comparing data obtained at 30 min and 1 h, Con A-induced IL-12 release capacity was significantly increased while both basal and PWM-induced IL-12 release capacities were significantly reduced (*p* < 0.001; **Figure 7**).

**FIGURE 8:**
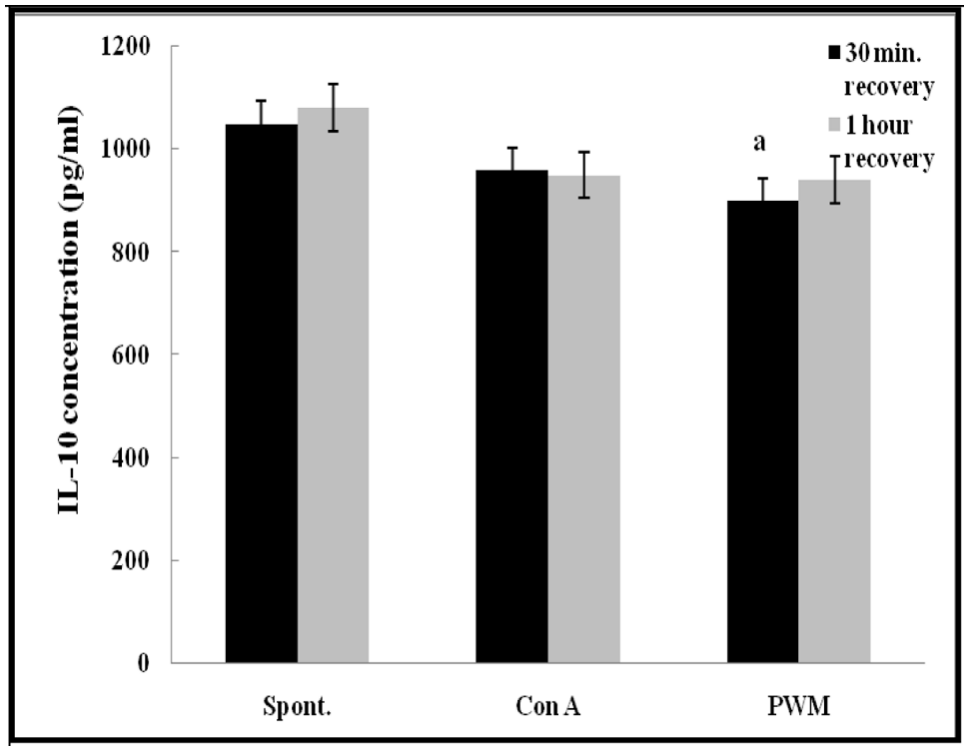
**IL-10 release either spontaneously or after *in vitro* stimulation with Con A or PWM in UVB irradiated lymphocytes suspension.** ^a^Level of significance between spontaneous and *in vitro* mitogen induced data.

Regarding IL-10 release capacity, after 30 min of recovery, there was a significant impairment in IL-10 release from PWM-stimulated cells as compared with un-stimulated cells (*p* = 0.028). However, no other significant changes were recorded in other comparisons either 30 min or 1 h of recovery after UVB irradiation (**Figure 8**).

## Discussion

Radiation is defined as the energy emitting from a source, moving through a space and absorbed by an object. It is classified according to the way they interact with normal chemical matter into ionizing and non-ionizing radiation. The non-ionizing spectrum is divided into two main regions; the electromagnetic fields and the optical radiations which can be further sub-divided into UV, visible and infra-red (Batten et al., 2008).

Ultraviolet radiation, particularly wavelengths in the UVB (290–320 nm) range, produces molecular changes in the skin that ultimately lead to marked deviations in the induction of cell-mediated immune responses by targeting antigen presenting cells (Nasti et al., 2011). UVB-induced damage of epidermal Langerhans cells, a subpopulation of DCs in the skin, is considered to be an important mechanism in UV-induced immunosuppression. Indeed, epidermal Langerhans cells are considered to be the principal targets of UV radiation as it inhibits their antigen-presenting activity and their capacity to stimulate Th1 cells (Vaid et al., 2013).

Accumulating evidences demonstrated the variability in responsiveness of different immune cells to radiations. Ionizing and non-ionizing radiations could shift the balance of Th1/Th2 cell response. This altered Th1/Th2 balance is attributed to modulation of cytokine- mediated transcriptional factors and signaling pathways. Hence enhanced Th2 response may mediate immune suppression and reduce cell mediated immunity (Yoshiki et al., 2012).

Our study focused on the effect of different durations of recovery on orienting the immune modulation induced by exposure to UVB radiation (in particular, cell proliferation and Th1/Th2 cytokine balance). Furthermore, elucidation of the influence of different forms of irradiation (direct exposure of lymphocytes versus whole body irradiation) was achieved. In addition, we tested the effect of adding of Con A and PWM on lymphocytes proliferation and cytokines release. It is well known that mitogens that originate from certain plants and bacteria induce mitosis and proliferation in large fractions (5–30%) of specific (B or T) lymphocyte subpopulations. Con A is a plant-derived lectin that directly stimulates peripheral T-lymphocytes to proliferate by binding non-specifically to numerous plasma membrane surface glycoproteins. Pokeweed mitogen is a plant lectin that preferentially stimulates B-cells but also induces some T-cell reactivity. Mitogen stimulation of lymphocytes *in vitro*, therefore, is used to evaluate general lymphocyte activity and detect differential proliferative responses in lymphocyte subpopulations (Vance et al., 2004).

Results obtained in the present study revealed that mice exposed to whole body UVB radiation exhibited suppressed lymphoproliferative response either spontaneously or after mitogen stimulation in comparison to un-exposed mice. There was a tendency recover by time although still below normal levels even after 6 days. Similar results were obtained when Wu et al. (2004) used the UVB to show that narrowband UVB irradiation stimulated a significant increase in the release of the basic fibroblast growth factor, by keratinocytes, which has been recognized as a natural mitogen for melanocytes.

Concerning IL-12 release, there was a significant suppression except for PWM-induced cells, followed by time dependent recovery that significantly exceeded the control levels especially when using Con A. These results may demonstrate that IL-12 can overcome UV-induced immune suppression. Results of IL-10 release showed significant elevation but this was broken after 7 days coinciding with IL-12 increase, suggesting that UVB exposure induces Th2 bias. This was in accordance with Rives and Ullrich (1994) who found that the activity of UV-irradiated T-cells was blocked with antibodies to IL-4 and IL-10, suggesting that these cells are Th2 cells. This was subsequently confirmed by Yagi et al. (1996) who cloned a suppressor cell from the spleen of UV-irradiated mouse that upon activation, it secreted IL-4 and IL-10.

Nasti et al. (2011) have reported that UVB exposure increases the production of the immunosuppressive mediators IL-10 and reduces the levels of immuno-stimulatory molecules like IL-12 and IL-23. They concluded that UV-induced expression of IL-10 contributes to the development of photocarcinogenesis by suppressing protective cellular immune responses of Th1 cells. Consistent with previously mentioned references, Sigmundsdottir et al. (2005) showed that PBMCs from UVB-treated psoriasis patients secreted greater amounts of IL-10.

*In vitro* exposure of lymphocytes to UVB radiation did not show remarkable effects on basal or mitogen-induced IL-10 after 30 min and 1 h recovery durations, while Con A-induced IL-12 was more elevated and spontaneous lymphocytes proliferation was reduced.

## Conclusion

Murine exposure to UVB radiation causes a state of immune suppression and shifts Th1/Th2 cell response. This effect is closely associated with the reduction of Th1 cytokines’ expression and increase in Th2 cytokines’ levels. This altered Th1/Th2 balance may be attributed to several factors including altered trafficking, functional behavior of antigen presenting cells, DNA damage-induced T-regulatory cells activities, and accumulated Cis-urocanic acid. The state of modulation of cell proliferation and cytokine release after UVB irradiation is largely dependent on source of irradiation, duration of exposure time, state of maturation of APC population, experimental design and target animal strain.

## Conflict of Interest Statement

The authors declare that the research was conducted in the absence of any commercial or financial relationships that could be construed as a potential conflict of interest.
